# Network analysis of autism traits and problematic mobile phone use and their associations with depression among Chinese college students

**DOI:** 10.3389/fpsyt.2024.1521453

**Published:** 2025-01-16

**Authors:** Gang Liu, Ya Liu, Zongping Chen, Siyuan Zhou, Lingfei Ma

**Affiliations:** School of Education Sciences, Chongqing Normal University, Chongqing, China

**Keywords:** autism traits, problematic mobile phone use, depression, network analysis, college students

## Abstract

The current study employed network analysis to examine the relationship between symptoms from factor level about autism traits and problematic mobile phone use (PMPU) and to explore their associations with depression. We measured the above three variables in 949 college students in China with Autism Spectrum Quotient (AQ), Smartphone Addiction Scale (SAS), Center for Epidemiological Studies Depression Scale (CES-D). Central and bridge symptoms were pinpointed through the examination of centrality index. In the AQ and PMPU network, results revealed that WD (“Withdrawal”), COR (“Cyberspace-oriented relationship”) and OU (“Overuse”) emerged as the core symptoms. AS (“Attention switching”), CO (“Communication”) and COR (“Cyberspace-oriented relationship”) were the most symptoms bridging the AQ and PMPU communities, suggesting that these symptoms could serve as focal points for interventions aimed at college students with concurrent autism traits and PMPU. SK (“Social skills”), COR (“Cyberspace-oriented relationship”), CO (“Communication”), and DLD (“Daily-life disturbance”) were most strongly associated with depression. In addition, future research should consider various measurement tools and methods to investigate the location of AD (“Attention to detail”), because AD was an isolated symptom in the flow network of depression.

## 1 Introduction

In individuals with autism spectrum disorder (ASD), deficiencies in social interaction, interpersonal communication, repetitive and restrictive behaviors and interests are characteristic features (1). These traits span from clinical to subclinical manifestations, initially identified in the immediate family members indicating genetic susceptibility (2). However, nowadays, personal and subclinical traits linked with autism have been demonstrated to be prevalent in the general population (3). Meanwhile, with further studies, researchers have found co-morbidity between clinical and sub-clinical symptoms and other disorder (4). Depression, anxiety, attention deficit hyperactivity disorder (ADHD), and substance-related addictive are typically the most common comorbidities, followed by several other disorders (5).

Addiction behaviors, especially problematic internet use, commonly occur in individuals with autism spectrum (6). Until now, there is strong evidence linking autistic symptoms with problematic internet use. For instance, individuals with ASD often show more severe symptoms of problematic internet use compared to those without ASD (7–9). Furthermore, college students or adults with higher autism traits are also at increased risk of developing problematic internet use (10, 11). On the one hand, this association could be attributed to rigid and limited behaviors, interests of the ASD phenotype (12). On the other hand, while the internet offers opportunities that assist individuals with ASD in overcoming offline challenges, their preoccupation with it may originate from limited interpersonal skills in face-to-face interactions (13, 14).

The link between autism traits and problematic mobile phone use (PMPU) has not received as much attention as problematic internet use. Here, we use the term “PMPU” because it is not formally classified in DSM-5 or ICD-11. PMPU involves excessive mobile phone use with addiction symptoms like tolerance, withdrawal, and continued use despite negative effects (15, 16). Despite mobile phones being common tools for internet access, PMPU is rising globally (17), with China notably affected (18). To date, few studies have explored the relationship between autism traits and PMPU. Research by Lu (19) suggested that elevated autism traits may increase the risk of PMPU among college students. Our research aims to investigate which aspects of autism traits are most associated with PMPU, paralleling previous research on problematic internet use.

Recent studies have shown that both autism traits and PMPU are associated with mental health issues, such as depression (20, 21). Higher levels of autism traits are linked to difficulties in face-to-face interactions, leading to challenges in interpersonal relationships and overall well-being, including increased depression (22). Similarly, PMPU has been consistently associated with psychological distress (e.g., depression) in various cross-sectional (23, 24) and longitudinal studies (25, 26). While some researchers proposed a unidirectional causal relationship between PMPU and depression (26, 27), others argued for a bidirectional association (28, 29). In summary, limited research has examined the concurrent impact of autism traits and PMPU, necessitating further exploration of their interaction with depression and underlying mechanisms.

Recent advances in methods allow us to use network analysis to understand mental disorders. In network modeling, mental syndromes and disorders are seen as intricate networks of symptoms that reinforce each other (30, 31). By measuring connections between symptoms, network models can identify key symptoms driving a disorder, thus pinpointing intervention targets (32). This approach overcomes the limitations of traditional psychopathology, which viewed symptoms as static constructs and potentially obscured important associations and distinctions. In network analysis, symptoms are nodes and their relationships are edges. Strength and expected influence (EI) are key centrality measures to identify pivotal symptoms (33). Bridge symptoms, those most linked with other conditions, offer insights into comorbid psychiatric mechanisms (34). Identifying these symptoms can uncover the underlying mechanisms of disorders and propose potential strategies for treatment. Network analysis has been applied to various concurrent psychiatric conditions, including autism spectrum disorder and internet addiction (35).

In this study, we aimed to: (1) analyze the network structure of autism traits and PMPU from factor level among Chinese college students in the general population; (2) identify central and bridge symptoms within the AQ and PMPU networks in non-ASD Chinese college students; (3) compare gender differences in AQ and PMPU network characteristics in the general population; and (4) identify symptoms directly and indirectly related to depression in the AQ and PMPU networks using the “flow” function. Given the exploratory nature of our study, specific hypotheses were not formulated. Our goal is to better understand the complex relationship between autism traits and PMPU, then offering insights that could inform interventions and support strategies for individuals with autism traits vulnerable to PMPU.

## 2 Methods

### 2.1 Participants

The study, conducted from September to October 2023 at a university in southwest China, aimed to investigate specific issues among university students. A total of 1029 questionnaires were distributed, resulting in 949 valid responses (436 males and 513 females), with a high effective response rate of 92.23%. The participants were from a general population of university students, spanning the ages of 19 to 23 (*M* = 19.12). Strict ethical principles were followed to protect participant privacy and rights throughout the data collection process.

### 2.2 Measures

Autism traits were measured using Chinese version of the Autism-Spectrum Quotient (AQ) tool (36), originally proposed by Baron-Cohen (37). The AQ included five factors, each factor with 10 questions, comprising social skills (e. g., “I find social situations easy”), attention switching (e. g., “New situations make me anxious”), attention to detail (e. g., “I often notice small sounds when others do not”), communication (e. g., “I am good at social chit-chat”), and imagination (e. g., “I find making up stories easy”). Each question was assessed ranging from 1 (definitely agree) to 4 (definitely disagree). A higher score on the AQ indicates a greater severity of autism traits. In this study, the Cronbach’s alpha was calculated to be 0.73.

The Smartphone Addiction Scale (SAS), introduced by Kwon (38), was utilized to measure the degree of problematic mobile phone use. With 33 items, the SAS encompassed six factors, comprising daily-life disturbance (e. g., “Missing planned works due to smartphone usage”), positive anticipation (e. g., “Feeling calm or cozy while using a smartphone”), withdrawal (e. g., “Won’t be able to stand not having a smartphone”), cyberspace-oriented relationship (e. g., “Feeling great meeting more people via smartphone use”), overuse (e. g., “Using my smartphone longer than I had intended”), and tolerance (e. g., “Always thinking that I should shorten my smartphone use time”). Each item was rated from 1 (“strongly disagree”) to 6 (“strongly agree”), with higher scores indicating greater severity. The Cronbach’s alpha was 0.93 in present study.

The subjects’ depression symptoms were surveyed by using the Center for Epidemiological Studies Depression Scale (CES-D), developed by Radloff (39). The scale comprised 20 items, rated on a scale between 1 (“rarely or none of the time”) and 4 (“most or all of the time”). Elevated total scores suggested greater severity of depression symptoms. In our study, the scale demonstrated strong internal consistency, with a Cronbach’s alpha of 0.91.

### 2.3 Data analysis

Before network estimation, Pearson’s correlation between factors was estimated using SPSS 27.0.

#### 2.3.1 Network estimation

The analysis was conducted using R-studio software. The study employed the Graphical Gaussian Model (GGM) to construct the network of autism traits and PMPU based on polychoric correlations (40). Each node in the network represents a specific symptom, connected by edges that indicate positive (blue) or negative (red) associations. Edge thickness (i.e. the edge weight) reflects the strength of the partial correlation coefficient. The network was refined using Lasso regularization (EBICglasso method) to optimize explanatory power by systematically reducing non-significant edges to zero. Visualization utilized the “qgraph” package, and node predictability was assessed using the “mgm” package (41). Additionally, the flow network exploring depression with autism traits and PMPU was estimated (42).

#### 2.3.2 Network centrality and stability

To evaluate node significance in the AQ and PMPU network, we used the “qgraph” package to analyze centrality indices: expected influence (EI; The sum of edge weights from the node to all other nodes including both positive and negative connections) (43); and bridge expected influence (bEI; The summed edge weights that a node to all other symptoms connecting two clusters of psychiatric symptoms) (34). Higher values of these indices indicate greater importance within the network. Subsequently, the accuracy and stability of network edges and centrality indices were assessed using the bootstrap method from the “bootnet” package (40).

#### 2.3.3 Network comparison

In line with earlier research suggestions, we explored variations in network attributes related to gender using the “NetworkComparisonTest” package. The Network Comparison Test (NCT) was employed to evaluate disparities, such as edge weight distributions, overall strength, and individual edges between networks based on gender, applying the Holm-Bonferroni correction for multiple test p-values (43).

## 3 Results

### 3.1 Correlation analyses

**Figure 1:**
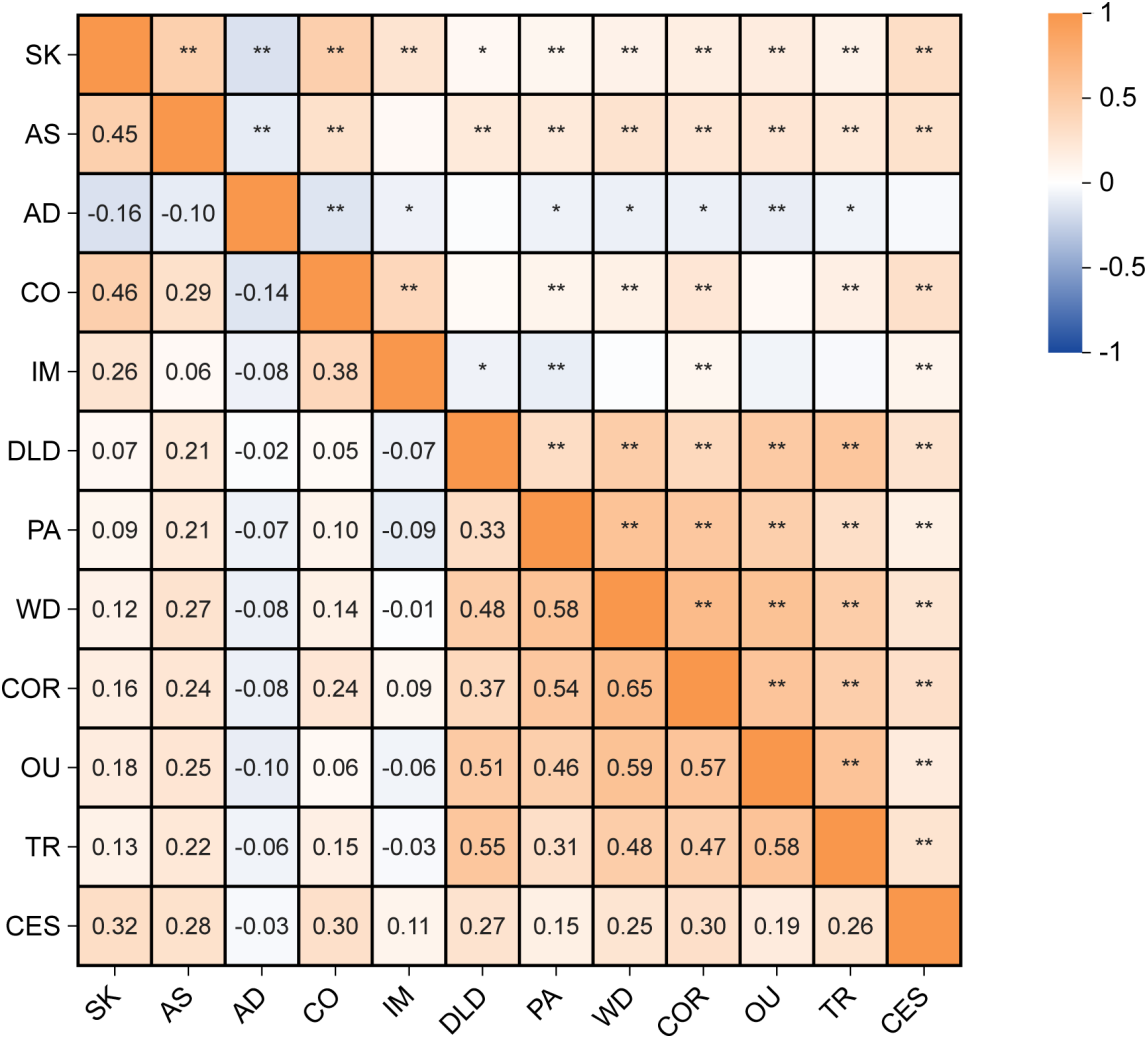
The correlation heat map of autism traits, problematic mobile phone use and depression. *p <.05, **p <.01. SK, Social skills; AS, Attention switching; AD, Attention to detail; CO, Communication; IM, Imagination; DLD, Daily-life disturbance; PA, Positive anticipation; WD, Withdrawal; COR, Cyberspace-oriented relationship; OU, Overuse; TR, Tolerance; CES, Depression.

Specific values of correlation analyses for the study variables are shown, accompanied by a correlation heat map (**Figure 1**) through the utilization of ChiPlot (https://www.chiplot.online/). Within the PMPU network, every symptom demonstrates a positive correlation with one another. Meanwhile, it is worth noting, one symptom (“Attention to detail”) and the other four symptoms have a negative correlation within the AQ network. In the whole network, contrary to correlation between three symptoms (“Attention switching”, “Communication”, “Social skills”) of AQ and each symptom of PMPU, two symptoms (“Attention to detail”, “Imagination”) of AQ and each symptom of PMPU have more negative edge.

### 3.2 Network structure

**Figure 2:**
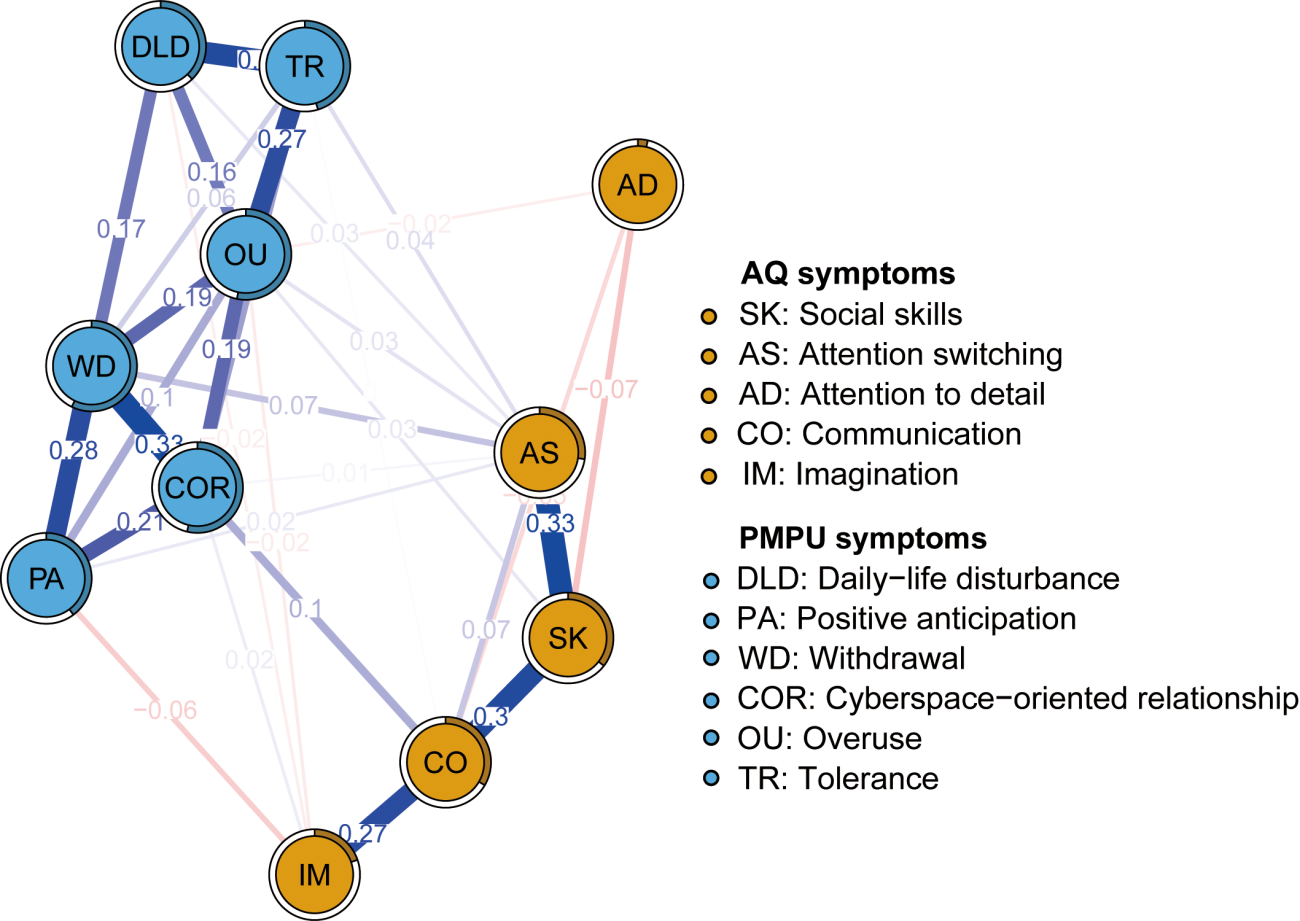
Network structure of autism traits and problematic mobile phone use.

**Figure 2** illustrates the network of AQ and PMPU symptoms among Chinese college students. The ring-shaped chart visually represents the predictability of individual symptoms, revealing an average predictability of 0.372. This suggests that neighboring nodes often explain approximately 37.2% of the variation in each node. Concrete predictability value of each node is shown in **Table 1**. In the symptoms of the AQ community, the strongest positive edge was between SK (“Social skills”) and AS (“Attention switching”), followed by the edges between SK (“Social skills”) and CO (“Communication”). In the PMPU community, the edge between COR (“Cyberspace-oriented relationship”) and WD (“Withdrawal”) was the strongest positive one, followed by the edges between nodes DLD (“Daily-life disturbance”) and TR (“Tolerance”). In the symptoms of the AQ and PMPU network, the strongest edge was CO (“Communication”) and COR (“Cyberspace-oriented relationship”), followed by the edges between AS (“Attention switching”) and WD (“Withdrawal”). The edge weights for the AQ and PMPU network can be found in **Supplementary Table S1**.

**Table 1:** Descriptive statistics of measurement factors.

Factor abbreviation	Factor content	Mean (SD)	Expected Influence	Predictability
SK	Social skills	2.45 (0.46)	1.754	0.355
AS	Attention switching	2.62 (0.30)	2.089	0.273
AD	Attention to detail	2.53 (0.38)	-0.903	0.035
CO	Communication	2.13 (0.36)	1.729	0.334
IM	Imagination	2.12 (0.33)	0.438	0.197
DLD	Daily-life disturbance	3.80 (1.02)	2.467	0.391
PA	Positive anticipation	3.47 (0.85)	2.441	0.407
WD	Withdrawal	3.37 (1.07)	3.221	0.572
COR	Cyberspace-oriented relationship	3.03 (0.90)	3.244	0.540
OU	Overuse	3.99 (1.01)	3.030	0.532
TR	Tolerance	3.56 (1.11)	2.787	0.454

**Figure 3:**
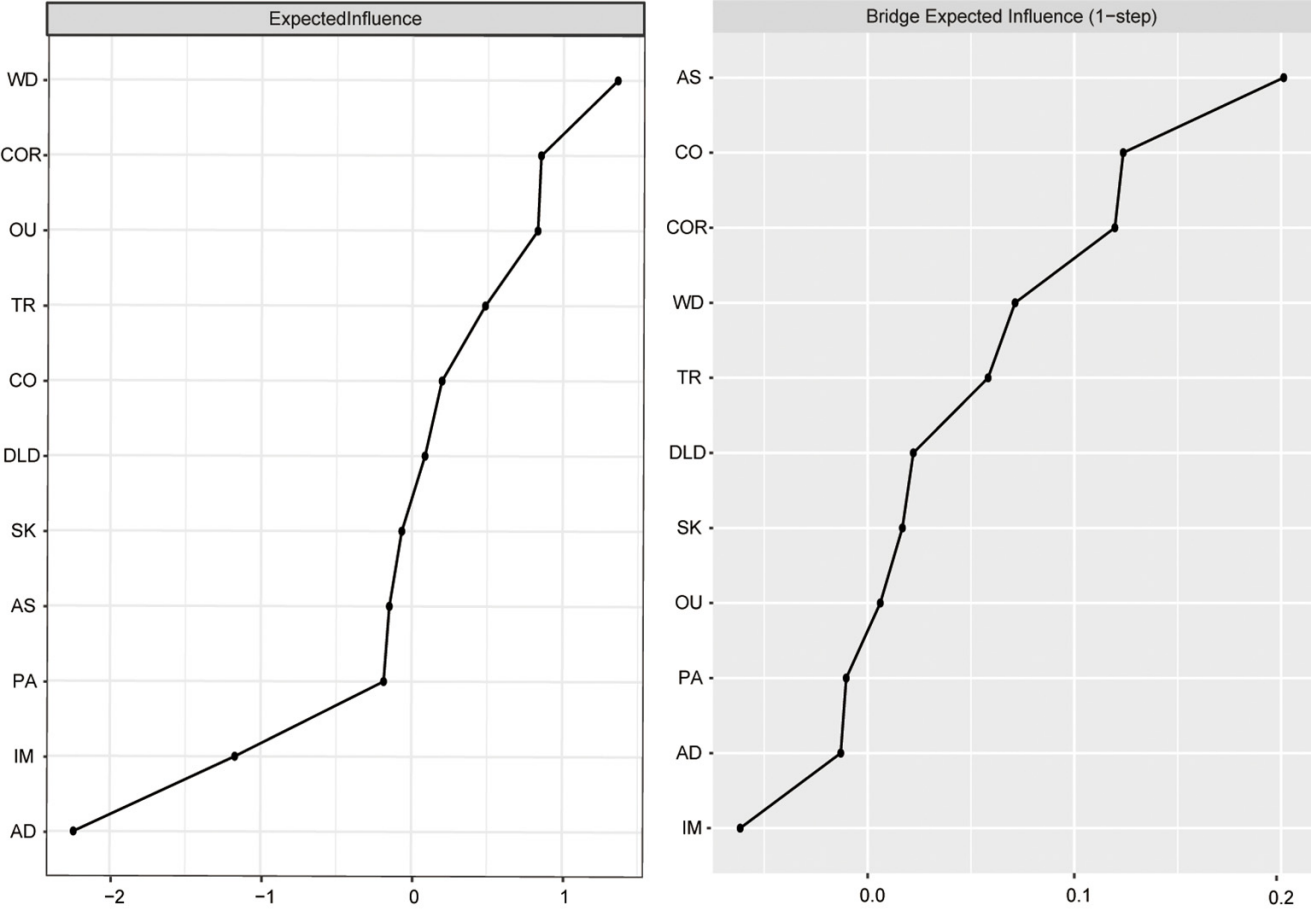
Centrality indices: EI and bEI values.

**Figure 3** shows the EI and bEI metrics of symptoms in the AQ and PMPU network. The most central symptom was WD (“Withdrawal”), followed by COR (“Cyberspace-oriented relationship”) and OU (“Overuse”) (**Figure 3**, left), implying these three symptoms are pivotal and exert significant influence in unraveling the framework of the AQ and PMPU network. The most core symptoms bridging the AQ and PMPU communities were AS (“Attention switching”), CO (“Communication”) and COR (“Cyberspace-oriented relationship”) (**Figure 3**, right part).

### 3.3 Network accuracy and stability

**Figure 4:**
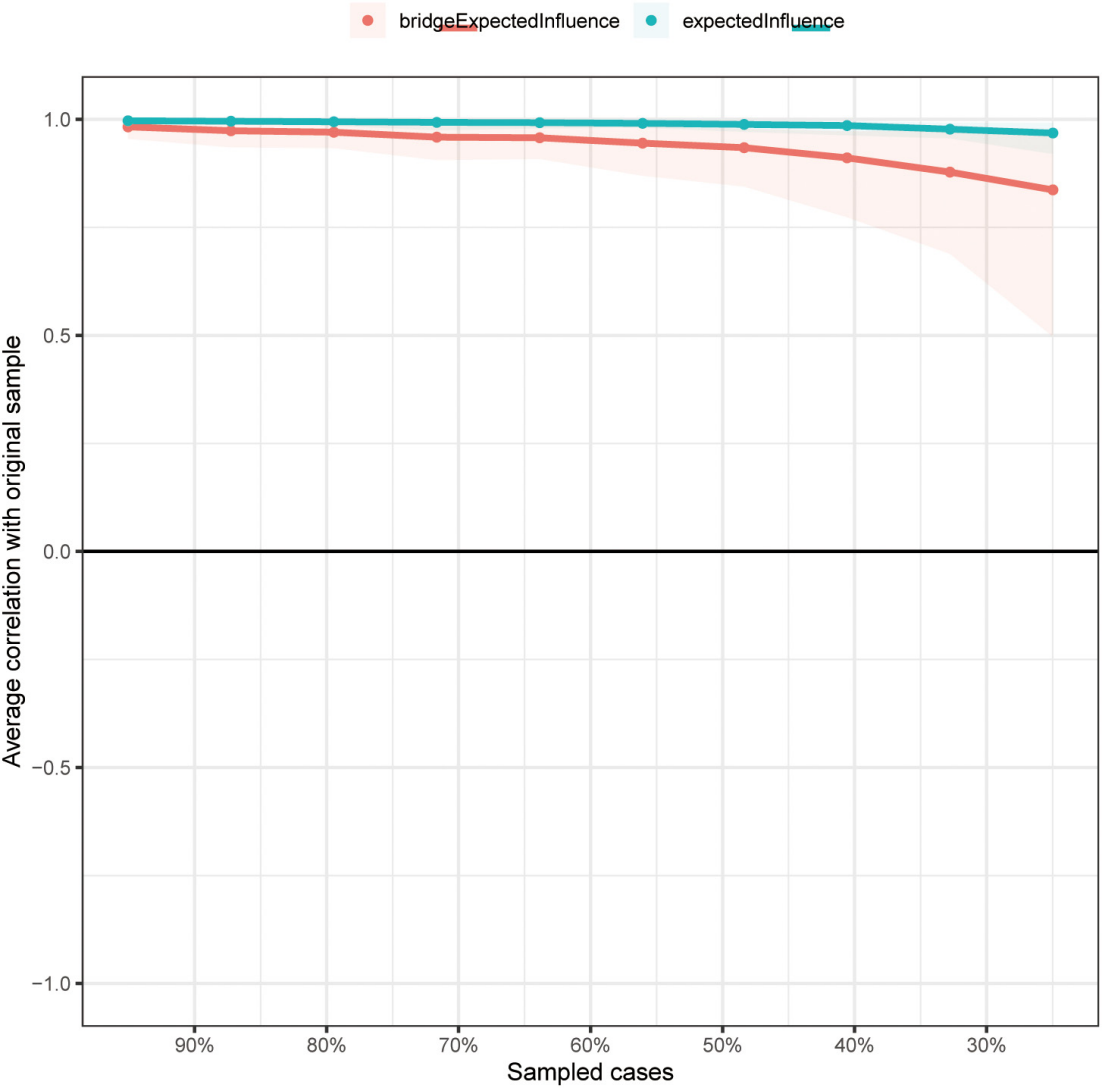
The stability of EI and bEI indices using case-dropping bootstrap.

At CS coefficients of 0.75 for EI and 0.672 for bEI, it indicates that when 75% or 67.2% of the sample are discarded, the EI and bEI networks will not change significantly (**Figure 4**). The outcomes of the bootstrap 95% CIs for edge weights by bootstrapped stability test are depicted in **Supplementary Figure S1**, and the findings of estimation of edge weight difference by bootstrapped difference test are illustrated in **Supplementary Figure S2**. According to the bootstrapped difference test for EI and bEI, the most influential nodes exhibited significant differences from the remaining symptoms (**Supplementary Figures S3**, **S4**).

### 3.4 Network comparisons based on gender

Previous findings indicated that the autism traits and PMPU levels had significant differences in gender among the general population, thus we compared network model between gender (3, 17). The network structure diagram of males and females is shown in **Supplementary Figure S5**. Analysis of network structures in male (n = 436) and female (n = 513) college students did not yield significant differences in network global strength (3.78 for males; 3.97 for females; S = 0.18, p = 0.655) or edge weights (M = 0.12, p = 0.570; **Supplementary Figure S6**).

### 3.5 Flow network of depression

**Figure 5:**
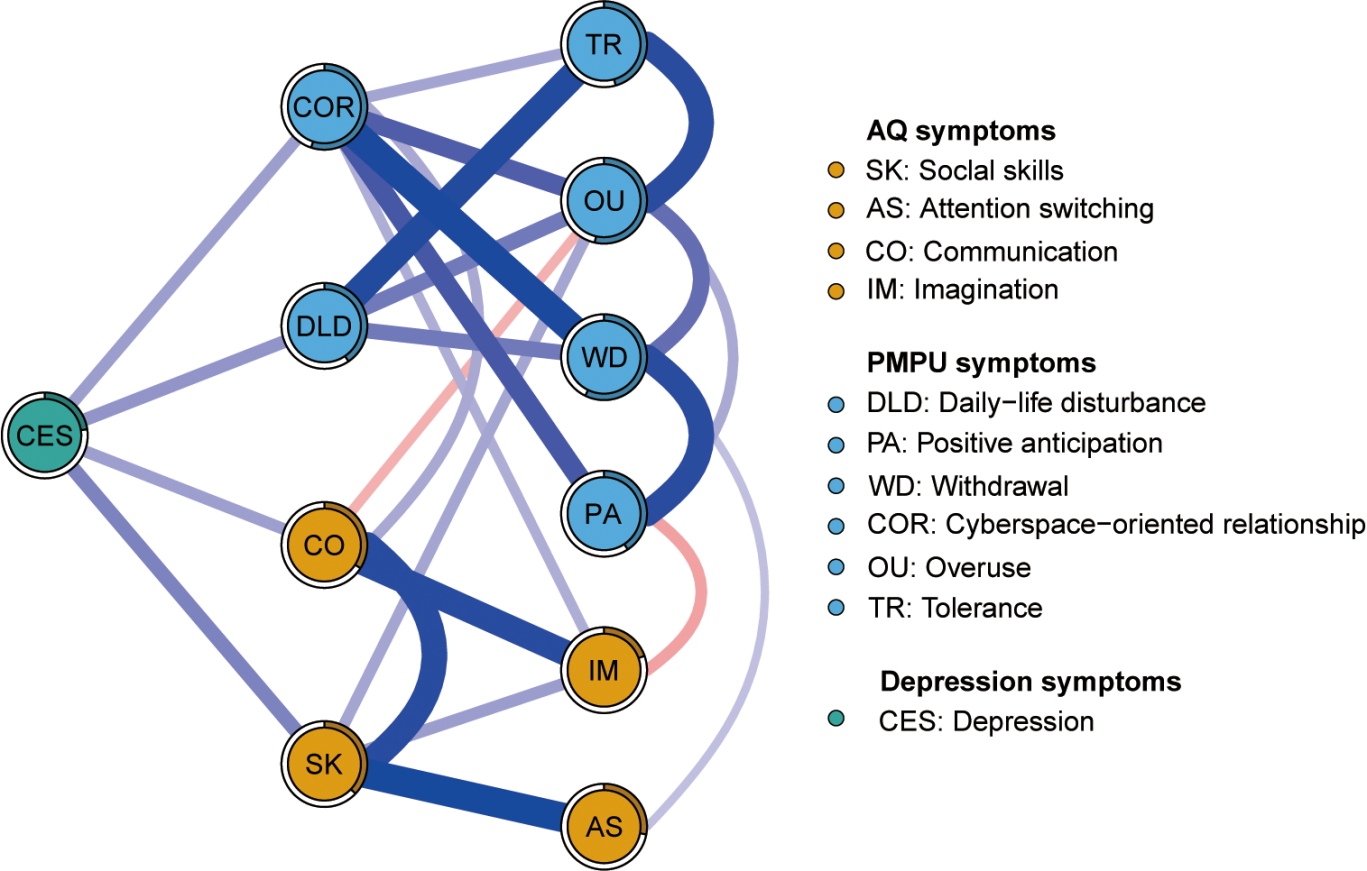
Flow network of depression.

The flow network diagrams about depression with autism traits and PMPU symptoms were created to explore which of their symptoms were related to depression (**Figure 5**). Because AD (“Attention to detail”) was identified as an isolated node, lacking connections to other nodes within these two networks, our study excluded it from the estimation of other symptoms. In the flow network of depression, the node SK (“Social skills”) emerged with the most robust positive correlation to depression, followed by the COR (“Cyberspace-oriented relationship”), CO (“Communication”), and DLD (“Daily-life disturbance”).

## 4 Discussion

To the best of our understanding, this investigation represents the initial exploration that probed into the network of AQ and PMPU symptoms from a factor structure level, and their association with depression in Chinese college students. In the AQ and PMPU network, the most central symptom was WD (“Withdrawal”), followed by COR (“Cyberspace-oriented relationship”) and OU (“Overuse”). These symptoms are very crucial for understanding the network structure of AQ and PMPU in the sample. Furthermore, the core ridge symptoms linking AQ and PMPU communities were AS (“Attention switching”), CO (“Communication”) and COR (“Cyberspace-oriented relationship”). We also noted that SK (“Social skills”), COR (“Cyberspace-oriented relationship”), CO (“Communication”), and DLD (“Daily-life disturbance”) were the most associated with depression. Notably, AD (“Attention to detail”) did not exhibit connections with other symptoms in the flow network of depression.

This was the first report to highlight its centrality in the AQ and PMPU network model. WD (“Withdrawal”) and OU (“Overuse”) were two of the central symptoms in this study. This finding aligns with the outcomes of an earlier investigation carried out among adolescents in Japan (35). They also found “Failure to cut down the time spent online” and “Staying online longer than you intend” were the central symptoms in the internet addiction network among a clinical and non-clinical sample with ASD. Extensive literature has reported excessive time use in both ASD and non-ASD individuals (9, 10, 44). These outcomes are consistent with addiction’s neurological pathways, often understood as a cycle of binging, withdrawal/negative affect, and preoccupation/anticipation (45). WD (“Withdrawal”) and OU (“Overuse”) correspond to the first two stages.

COR (“Cyberspace-oriented relationship”) was another central symptom indicating that individuals with higher autism traits prefer interaction through mobile phones over real-life situations. Social interaction’s significance in the relationship between PMPU and autistic traits could explain outcomes (19). Individuals with higher autism traits often face challenges in interpersonal interactions due to inhibitions and perceived incompetence, making them vulnerable. Despite their aversion to direct social interactions, there is no clear association with a desire for social exclusion. As such, they may gravitate towards the safer realm of online communication, which offers them opportunities to address these challenges (13, 14). This preference for online interactions could strain traditional social bonds over time, possibly contributing to addiction to online games as a means of avoiding real-life interaction.

The bridge symptoms, AS (“Attention switching”), CO (“Communication”) and COR (“Cyberspace-oriented relationship”), should be central targets for therapeutic interventions addressing PMPU in individuals with higher autism traits. AS (“Attention switching”) was the most core bridge symptom, suggesting that attention deficiency is a key factor in PMPU symptoms among these individuals. One possible explanation is that ADHD and autism traits have strong comorbidities in genes, neurobiology, and behaviors (46), with related symptoms extending into adulthood (47). While not confirmed across the broader population, individuals with ASD exhibit a higher risk for problematic internet use and ADHD symptoms (44). Additionally, cognitive deficits like inhibition control may cause difficulty in controlling internet use once engaged.

Another two key bridge symptoms were CO (“Communication”) and COR (“Cyberspace-oriented relationship”). Difficulties in communication and social interaction in individuals with higher autism traits interact with problematic internet use (10, 11). However, the modes of communication and interaction among individuals with higher autism traits are not entirely clear. A recent study found individuals with ASD using electronics for fewer social activities than general population while they engage in face-to-face communication under protest (7). The result may seem counterintuitive. Yet, interestingly, the preference for communication for individuals with ASD is dependent upon how close and accepting the relationship is (48). In consideration of complex modes of communication, future studies should compare communication styles between individuals with higher autism traits and those with ASD to improve communication levels (49).

In the flow network, SK (“Social skills”), COR (“Cyberspace-oriented relationship”), CO (“Communication”), and DLD (“Daily-life disturbance”) showed the strongest associations with depression compared to other symptoms. SK (“Social skills”) was found to have the strongest association with depression, consistent with recent research linking social ability to increased depression (22). Individuals with social deficit showed reduced risk avoidance, heightening susceptibility to frustration and depression. COR (“Cyberspace-oriented relationship”) and CO (“Communication”) showed the second strongest association with depression, highlighting that while smartphones can compensate for offline communication deficits, excessive use may exacerbate negative emotions (26, 28). DLD (“Daily-life disturbance”) ranked third in association with depression. Previous studies indicated that depression negatively impacts sleep quality, leading to heightened stress and affecting overall life satisfaction (26, 28).

Notably, AD (“Attention to detail”) emerged as an independent symptom in the flow network, showing no significant associations with other symptoms. Correlation analyses across the AQ and PMPU network similarly revealed a negative or zero correlation with other symptoms. This aligns with autism diagnostic criteria, which distinguish between social and non-social domains. AD (“Attention to detail”) represents a non-social dimension within the AQ scale used in general population studies (50). However, our findings diverge from previous research indicating no positive association between internet preoccupation and repetitive behaviors in college students (12). This discrepancy suggests a need for careful interpretation, possibly influenced by measurement limitations in assessing Autism-Spectrum Quotient traits.

Our findings underscore the importance of targeting bridge symptoms such as AS (“Attention Switching”), CO (“Communication”), and COR (“Cyberspace-oriented relationship”) in designing therapeutic interventions for individuals with higher autism traits experiencing PMPU. Interventions that enhance attention regulation, such as mindfulness training or cognitive-behavioral therapy (CBT) focused on improving executive functioning, could be particularly effective in mitigating PMPU symptoms related to attention deficits (14). Similarly, social skills training programs could address CO (“Communication”) challenges by equipping individuals with practical strategies for face-to-face interactions, reducing reliance on cyberspace relationships (35). For COR (“Cyberspace-oriented relationship”), interventions might include psychoeducation on balanced technology use and guided exposure to real-life social interactions to build confidence and resilience (19). These approaches could reduce the negative emotional impacts associated with PMPU, such as depression, while also promoting healthier social and behavioral patterns. Future research should evaluate the efficacy of such interventions to further refine these strategies and ensure their applicability.

This study involves several limitations. Firstly, it does not clarify the similarities and differences in PMPU between individuals with ASD and those without ASD, as it primarily focuses on the general population. Future studies should aim to include samples from both groups. Secondly, symptoms are not entirely specific at the factor level due to the inclusion of multiple items within a single factor. Finally, our findings provide only a preliminary exploration of the overlapping symptoms between autism traits and PMPU. Given the varied motivations for smartphone use, these overlapping symptoms may differ significantly. Therefore, further research should consider specific aspects of problematic mobile phone use, such as video consumption, gaming, or social media engagement, to provide a more comprehensive understanding.

## Data Availability

The raw data supporting the conclusions of this article will be made available by the authors, without undue reservation.
