# Supplementary material for: Network analysis of autism traits and problematic mobile phone use and their associations with depression among Chinese college students
Source: Front Psychiatry. 2025 Jan 16;15:1521453. doi: 10.3389/fpsyt.2024.1521453 (PMC11779719; doi:10.3389/fpsyt.2024.1521453)
Supplement: Supplementary file 1 [file SupplementaryFile1.docx]

**Supplementary Materials**

Table S1. Partial correlation matrix of the AQ and PMPU symptoms

Figure S1. Bootstrapped confidence intervals of edge weights

Figure S2. Estimation of edge weight difference by bootstrapped difference test

Figure S3. Estimation of ExpectedInfluence difference by nonparametric bootstrapped difference test

Figure S4. Estimation of bridgeExpectedInfluence difference by nonparametric bootstrapped

Figure S5. Estimated network model for the association between autism trait and problematic mobile phone use in females and males

Figure S6. Comparison of network properties between females and males

Table S1. Partial correlation matrix of the AQ and PMPU symptoms

|  | SK | AS | AD | CO | IM | DLD | PA | WD | COR | OU | TR |
| --- | --- | --- | --- | --- | --- | --- | --- | --- | --- | --- | --- |
| SK | 0.000 |  |  |  |  |  |  |  |  |  |  |
| AS | 0.317 | 0.000 |  |  |  |  |  |  |  |  |  |
| AD | -0.055 | 0.000 | 0.000 |  |  |  |  |  |  |  |  |
| CO | 0.274 | 0.062 | -0.026 | 0.000 |  |  |  |  |  |  |  |
| IM | 0.054 | 0.000 | 0.000 | 0.258 | 0.000 |  |  |  |  |  |  |
| DLD | 0.000 | 0.022 | 0.000 | 0.000 | 0.000 | 0.000 |  |  |  |  |  |
| PA | 0.000 | 0.024 | 0.000 | 0.000 | -0.034 | 0.009 | 0.000 |  |  |  |  |
| WD | 0.000 | 0.071 | 0.000 | 0.000 | 0.000 | 0.146 | 0.280 | 0.000 |  |  |  |
| COR | 0.000 | 0.009 | -0.013 | 0.124 | 0.000 | 0.000 | 0.190 | 0.307 | 0.000 |  |  |
| OU | 0.017 | 0.017 | 0.000 | 0.000 | -0.028 | 0.193 | 0.100 | 0.184 | 0.168 | 0.000 |  |
| TR | 0.000 | 0.058 | 0.000 | 0.000 | 0.000 | 0.285 | 0.000 | 0.079 | 0.118 | 0.244 | 0.000 |

Figure S1. Bootstrapped confidence intervals of edge weights

Figure S2. Estimation of edge weight difference by bootstrapped difference test

Figure S3. Estimation of ExpectedInfluence difference by nonparametric bootstrapped difference test

Figure S4. Estimation of bridgeExpectedInfluence difference by nonparametric bootstrapped difference test


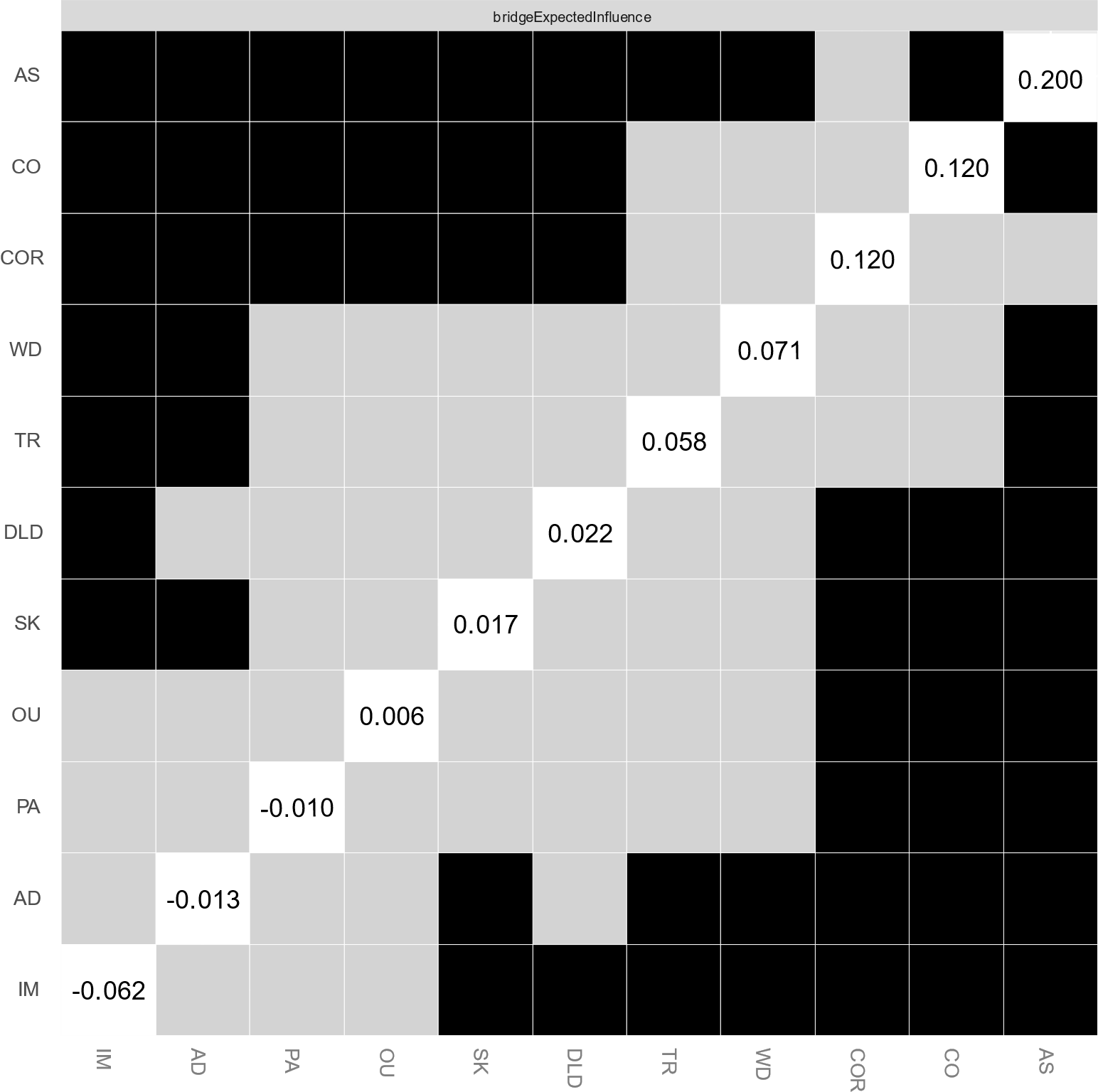


Figure S5. Estimated network model for the association between autism trait and problematic mobile phone use in females and males

Figure S6. Comparison of network properties between females and males


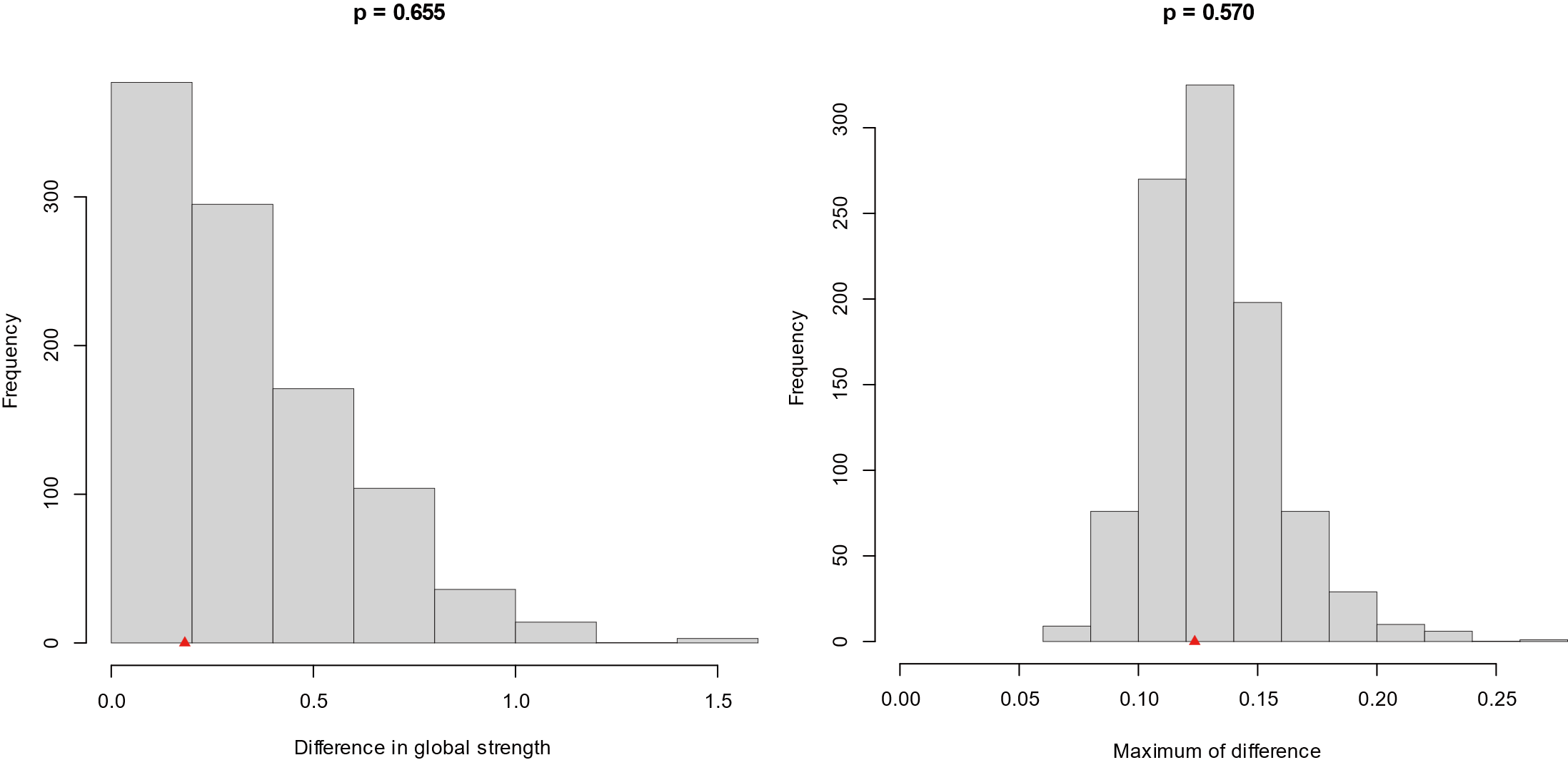


Figure S6. Left Panel: A plot of bootstrap value of the difference in network global strength. The difference was not significant (network strength among male participants: 3.78; among female participants: 3.97; S: 0.18, p = 0.655). Invariance in edge weights was tested using the permutation test, generating sets of p values for each edge-edge comparison. Holm-Bonferroni corrected p values were all > 0.05 indicating absence of significant differences.

Right Panel: A plot of bootstrap value of the maximum difference in any of the edge weights (1000 permutations). The difference was not significant (M = 0.12, p = 0.570)
